# Subcapsular double-J stent following ureteroscopy: unique complication

**DOI:** 10.1093/jscr/rjaa404

**Published:** 2020-10-19

**Authors:** Ahmed Aljuhayman, Faisal Balaraj, Yahya Ghazwani, Saeed Bin Hamri

**Affiliations:** Department of Urology, King Abdulaziz Medical City, Riyadh, Saudi Arabia; King Abdullah International Research Center, Riyadh, Saudi Arabia; Majmaah University, College of Medicine, Al Majma'ah, Saudi Arabia; Department of Urology, King Abdulaziz Medical City, Riyadh, Saudi Arabia; King Abdullah International Research Center, Riyadh, Saudi Arabia; King Saud bin Abdulaziz University for Health Sciences, College of Medicine, Riyadh, Saudi Arabia; Department of Urology, King Abdulaziz Medical City, Riyadh, Saudi Arabia; King Abdullah International Research Center, Riyadh, Saudi Arabia; King Saud bin Abdulaziz University for Health Sciences, College of Medicine, Riyadh, Saudi Arabia; Department of Urology, King Abdulaziz Medical City, Riyadh, Saudi Arabia; King Abdullah International Research Center, Riyadh, Saudi Arabia; King Saud bin Abdulaziz University for Health Sciences, College of Medicine, Riyadh, Saudi Arabia

**Keywords:** retrograde pyelography, double-J ureteral stent, endourological complications

## Abstract

Double-J (DJ) ureteral stent is a standard procedure in daily urological practice performed to relive ureteral obstruction or as a part of other endourological procedures. Although it is a common procedure, the widespread use of ureteral stents has corresponded to the increase in possible complication. We report a unique complication for a patient who presented with a renal subcapsular complete misplacement of DJ stent postureteroscopy for a distal ureteric stone. This challenging complication of ureteral stents is rare and organ threatening.

## INTRODUCTION

Double-J (DJ) ureteral stent is a standard procedure in daily urological practice performed for the relief of ureteral obstruction or as a part of other endourological procedures; nevertheless, the widespread use of ureteral stents has corresponded to the increase in possible complications related to stent insertion such as encrustation and misplacement of ureteric stent, subcapsular hematoma and infections [1].

We report the first case of unique complication for a patient who presented with a renal subcapsular complete misplacement of DJ stent postureteroscopy (URS) for a distal ureteric stone. This challenging complication of ureteral stents is rare and organ threatening.

## CASE

**Figure 1:**
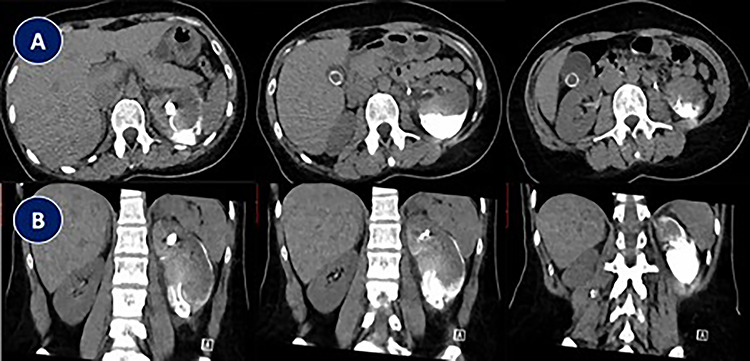
Abdominal CT scan showing complete misplacement of DJ stent into subcapsular renal space. (**A**) CT scan image (Axial). (**B**) CT scan image (Coronal).

This is a 33-year-old female with no co-morbid conditions, presented to another center with severe left flank pain for 3 days associated with nausea and vomiting. She had abdominal non-contrast CT showing 3-mm left distal ureteric stone. Patient was treated with medical expulsive therapy, which did not work for 14 days, and patient came to ER twice during that period. Therefore, patient underwent left ureteroscopy, during the procedure stone was removed, decision was to insert a DJ stent. According to the urologist, DJ stent was misplaced completely proximally. Therefore, he inserted another DJ stent. Patient was in severe pain postoperatively; abdominal CT scan that was carried out showed complete misplacement of DJ stent into subcapsular renal space [Fig. 1]. After 1 week, patient was referred to our center and admitted due to persistent left flank pain associated with intermittent gross hematuria. Physical examination was unremarkable apart from left flank tenderness. Her blood investigations showed: WBC: 7.87 × 10^9^/L, RBC: 3.86 × 10^9^/L, Hgb: 10.4 g/dl and creatinine 50 μmol/L. Urine analysis showed: RBC: 56, WBC: 79 and nitrate was negative. Urine culture showed negative growth for organisms. Abdominal non-contrast CT was performed in our center; images were discussed with senior radiologist who could not confirm if the tip of misplaced subcapsular renal DJ stent was in the left upper calyx; however, he confirmed adequately positioned second left DJ stent.

**Figure 2:**
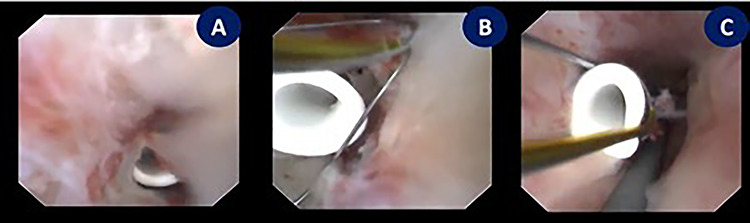
Endoscopic exploration revealing the tip of a subcapsular renal DJ stent intraparenchymal just behind the renal papilla, managed by using N-Gage from (Cook) in order to confront-capture the DJ stent tip. (**A**) Tip of a DJ stent intraparenchymal. (**B** and **C**) N-Gage basket capturing the tip of a DJ stent.

**Figure 3:**
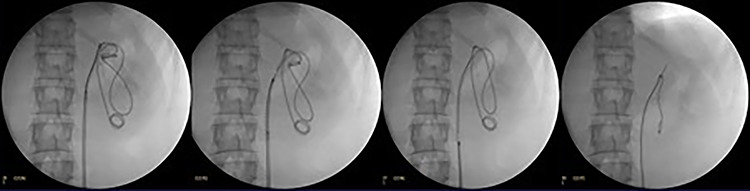
Fluoroscopic images showing DJ stent removal in steps.

As it was shown in CT scan, most of the DJ stent was subcapsular, patient was counseled regarding percutaneous approach, laparoscopic approach, or flexible ureteroscopy with possibility to use the previously mentioned approaches if needed. Patient elected to go for flexible ureteroscopy.

Patient underwent under general anesthesia. Cystoscopy was done, after that two guide wires, one for safety and the other one for ureteric access sheath were inserted under fluoroscopic guidance. Ureteral access sheath (UAS) was used to reduce the intrarenal pressure and by that we aimed to reduce the extravasation. Ureter was intact on retrograde pyelogram with no extravasation. A 35-cm 10/12F ureteric access sheath was passed over the guide wire and nephroscopy started. The endoscopic exploration revealed that the tip of subcapsular renal DJ stent was intraparenchymal just behind the renal papilla; therefore, we used N-gage from Cook in order to confront-capture the DJ stent tip [Fig. 2]. DJ stent was removed smoothly as shown in fluoroscopy [Fig. 3]. Patient was discharged in day one postoperatively. Patient was doing fine till we saw her 6 weeks postremoval of DJ stent not complaining of any symptoms except small perinephric collection on renal ultrasound. That collection completely disappeared in 6 months of follow-up.

## DISCUSSION

DJ ureteral stents have been a standard procedure and widely used in urological practice. DJ ureteral stents have been used to establish internal drainage after endourological/reconstructive procedures; however, significant complications are common and may occur from inserting a ureteral stent such as misplacement, migration, encrustation, subcapsular hematoma and infection.

According to our literature search, Dundar et al. [1] was the first to report case with a renal parenchymal perforation with a DJ stent in a patient with a solitary kidney. DJ stent was placed after a ureteroscopy; thereafter, patient was unstable and developed atrial fibrillation with a rapid ventricular response and anuria. CT scan was done showing that the DJ stent was found to be out of the collecting system, with a perforation of the renal parenchyma and a subcapsular hematoma. Patient was managed by repositioning of the DJ stent under fluoroscopy guidance as well as blood transfusion.

Nomikos et al. [2] reported a case of a 62-year-old female patient with a solitary functioning kidney presented with a left-sided two ureteral stones when DJ stent was inserted. Postoperatively, patient was complaining of left-sided abdominal tenderness and gross hematuria; thereby, non-contrast abdominal CT scan was performed showing a 12 × 8-cm perirenal hematoma and the tip of the DJ stent penetrating the parenchyma without any contrast extravasation. A week after, patient was also managed by repositioning the DJ stent under fluoroscopic guidance.

Pradhan et al. [3] reported a case of a 62-year-old female known to have carcinoma of the cervix treated with chemo-radiation, patient presented with intermittent gross hematuria, which was managed by a DJ stent as there was a suspicion of a right ureteral stricture; 3 months later DJ stent was exchanged for the previous placed stent. Both procedures were done without fluoroscopy guidance. Postoperatively, patient presented with three episodes of febrile UTI; to evaluate the cause of her febrile illness, a CT scan was done, which revealed an extrusion of the upper end of the double J stent outside the renal parenchyma. The patient was managed by removal of the stent by cystoscopy, and a retrograde was done showing normal ureter; hence no stent was inserted.

Altay B et al. [4] reported a case of a 36-year-old female, who presented with a (9-mm) obstructive proximal ureteric stone, which was managed by ureteroscopic lithotripsy and 4.6F DJ stent insertion. On postoperative day 2, patient presented with sever flank pain; therefore, abdominal CT was done showing that the proximal tip of DJ stent perforated the renal parenchyma and curled in the perirenal area. Patient was managed under local anesthesia, with cystoscopy where the DJ stent was grasped distally with a foreign substance forceps under fluoroscopic guidance and brought back into proximal collecting system.

Gönülalan et al. [5] reports a case of a 66-year-old male who presented to their clinic with left flank pain and hematuria. Patient underwent DJ stenting at another medical center due to secondary obstruction due to left ureteropelvic junction obstruction due to prior pyeloplasty operation. CT done revealed a renal parenchymal perforation was caused by the DJ stent, posterior perirenal extravasation and reactive pleural effusion. Patient underwent repositioning of the DJ stent in the renal pelvis under fluoroscopic guidance. Patient was discharged second postoperative day with no complication.

Our case is unique and to our knowledge, it is the first case in literature with an unusual event of a subcapsular complete misplacement of DJ stent post-URS. This complication is challenging, management of such a case needs a high clinical decision whether to be treated endoscopically, laparoscopically or by other valid options such percutaneous access. Our case was managed endoscopically, and the patient was discharged day one postoperatively in good condition. In conclusion, insertion of a DJ stent should be performed carefully. This case should remind the endourologist about the serious complications following implantation of a DJ stent, which is used frequently worldwide.
